# Mechanism of Huangqi Sanxian Decoction Inhibiting Osteoclast Differentiation Based on Network Pharmacology

**DOI:** 10.1155/2022/8769531

**Published:** 2022-06-08

**Authors:** Jian-Xin Shi, Xiao-Qun Cai, Ze-Hao Zhao, Yu Deng, Zhi-Kun Zhou

**Affiliations:** ^1^Department of Pharmacy, Guangdong Medical University, No. 1, Xincheng Dadao, Songshan Lake Science and Technology Industry Park, Dongguan 523808, China; ^2^Huizhou Women's and Children's Hospital Affiliated to Guangdong Medical University, No. 10, Daling Road, Huicheng District, Huizhou 516001, China

## Abstract

Osteoclasts (OCs) have been the unique cell type exhibiting the bone-resorption activity in body. It is important to identify drugs to resist osteoclastogenesis to manage the bone-loss disorders. Huangqi Sanxian decoction (HQSXD) is utilized for the treatment of postmenopausal osteoporosis (PMOP) for a long history in East Asia. This work aimed to examine HQSXD's activity in OC differentiation. Based on staining with tartrate-resistant acid phosphatase (TRAP), it was found that HQSXD suppressed OC generation under the induction of RANKL produced in the bone marrow-derived monocytes/macrophages (BMMs), with no cytotoxic effect. Later analysis like molecular exploration and network pharmacology (NP) suggested the role of HQSXD in suppressing genes associated with osteoclastogenesis via PI3K/Akt-mediated mechanism dose-dependently. This work might illustrate the molecular pharmacological mechanism involved in HQSXD's effect on treating OC-associated disorders. Moreover, NP was found to modernize traditional Chinese medicine (TCM) research.

## 1. Introduction

Bone is constituted by minerals and proteins. In a mature skeletal system, the dynamically balanced bone formation-bone resorption can maintain persistent bone tissue metabolism, thereby maintaining bone elasticity and stiffness [1,2]. Osteoclasts (OCs) are the unique cells that allow for bone resorption in body, and bone marrow monocytes/macrophages (BMMs)-derived OCs have critical effects on bone remodeling [3]. However, when OCs have excessively high activity, it may result in various osteopathic disorders, like osteoporosis (OP), rheumatoid arthritis (RA), and bone tumor [4–6].

Macrophage colony stimulating factor (M-CSF) and receptor activator of nuclear factor-*κ*B (NF-*κ*B) ligand (RANKL) can be generated via osteoblasts (OBs) or additional bone cells, which have critical roles in OC generation [7,8]. Of them, M-CSF can activate the corresponding receptors on the BMM surface and offer the signals required by precursor OC growth [9]. On the other hand, RANKL can combine with the receptor activator of NF-*κ*B (RANK), and it has the major functions of inducing myeloid precursor differentiation to mature OCs [10]. The BMMs-derived mature OCs under the induction of M-CSF and RANKL are recognized through staining with tartrate-resistant acid phosphatase (TRAP) in vitro, which can produce resorption pits onto bone fragment surfaces for achieving bone resorption [11,12]. Additionally, RANKL can trigger diverse transcription factors (TFs), like nuclear factor of activated T-cells, cytoplasmic 1 (NFATc1) as well as c-Fos, thus up-regulating various specific gene expression like matrix metalloproteinase-9 (MMP-9), TRAP, cathepsin K, and ATPase H + transporting V0 subunit d2 (ATP6v0d2) [13–15]. The abovementioned genes and TFs are tightly associated with OC generation and activity, which are thus extensively utilized for assessing osteoclastogenesis.

In the theory of traditional Chinese medicine (TCM), the kidney is suggested to regulate bone activities. A potent “kidney” is indicated to nourish the bones, on the contrary, an impotent “kidney” may aggravate bone degeneration [16]. Blood stasis and kidney deficiency have laid the major pathological foundation for OP. Huangqi Sanxian decoction (HQSXD) consists of 8 herbal medicines, among which, *Radix notoginseng* and *Radix salviae miltiorrhizae* can promote blood circulation, whereas *Cistanche herba* and *Epimedii folium* have kidney-strengthening activity. The above herbal medicines account for a superb combination that can highlight the corresponding superiority in treating OP [17].

In this study, the findings provide a rationale for using HQSXD to treat OP in clinic.

## 2. Materials and Methods

### 2.1. Animals

This work obtained 50 Sprague-Dawley (SD) rats (females), weighing approximately 100–150 g in the Animal Experiment Center of Guangdong Medical university (Dongguan, China). Afterwards, each rat was raised under 24 ± 0.5°C and 12 h/12 h light dark cycle conditions (light on: 08 : 00–20 : 00), with free access to water and food throughout the experiment. After 7 days of acclimatization, we randomized these rats in 5 groups, including blank control, raloxifene-treated positive control, and Huangqi Sanxian decoction (containing high and middle and low concentrations)-treated experimental groups. The present work gained approval from Laboratory Animal Management Committee. Each experiment was carried out following relevant regulations and rules.

### 2.2. Preparation of HQSXD

HQSXD consisted of 8 plant extracts, namely, *Radix astragali* (Huang-Qi in Chinese herbal name; root), *Epimedii folium* (Yin-Yang-Huo; leaf), *Cistanche herba* (Rou-Cong-Rong; succulent stem), *Radix notoginseng* (San-Qi; rhizome and root), Radix salviae miltiorrhizae (Dan-Shen; rhizome and root), *Corydalis rhizoma* (Yan-Hu-Suo; rhizome), *Radix angelicae Sinensis* (Dang-Gui; root), as well as *Radix clematidis* (Wei-Ling- Xian; rhizome and root) at the 15 : 10 : 10 : 5 : 10 : 10 : 8 : 10 ratio [6]. This work acquired these 8 extracts from Dongguan Sinopharm (Dongguan, China). Meanwhile, Professor Zhou (Department of Pharmacy, Guangdong Medical College, Dongguan, China) was invited to identify these extracts. All the abovementioned 8 extracts were mixed to yield a 270 g mixture, which was later ground into powders, followed by 2 h boiling with water (1000 mL) twice. Thereafter, at reduced pressure, we concentrated the filtrates to 200 mL and maintained them under 4°C.

### 2.3. Drug Administration and Sample Collections

This work randomized female rats as 5 groups, with 10 in each group. Mice were given oral administration of HQSXD solution (High: 27.501 g/kg; Middle: 9.167 g/kg; Low:3.056 g/kg) and Raloxifene solution (6.25 g/kg) once daily for 7 consecutive days, whereas those in normal group were given oral administration of distilled water and monitored at the same time of those in experimental groups. At 60 min post the final treatment, each rat was anesthetized to collect blood through heart puncture aseptically. After 15 min centrifugation of blood, sera were obtained from rats of the above 3 groups, followed by 30 min inactivation under the 56°C water bath as well as filtration with the 0.22 *μ*m membrane, so as to obtain HQSXD (H)-S, HQSXD (M)-S, HQSXD (L)-S, Ral -S, as well as Normal-S, separately, which were preserved under −80°C.

### 2.4. Primary Osteoclast Culture and CCK-8 Assay

Primary rat osteoclast cells were obtained from bone medullary cavity of 1-Month-old neonatal Sprague-Dawley rats with 20 ng/mL M-CSF Induced. For investigating how HQSXD toxicity affected BMMs' viability, this work cultivated BMMs (1 × 10^6^/well) into the 96-well plate, followed by M-CSF incubation. On day 2, HQSXD at specific doses was utilized for 24-, 48-, and 72-h cell treatment. Cell viability was detected by CCK-8 assay. Afterwards, all wells were added with CCK-8 solution (10 *μ*l), followed by another 1-h plate incubation. The infinite F200 PRO absorbance microplate reader (Tecan Group Ltd., Mannedorf, Switzerland) was employed for measuring the absorbance (OD) values at 450 nm (OD450). Finally, this work determined cell viability based on control.

### 2.5. TRAP Colorimetric Assay

This work inoculated BMMs (3 × 10^6^/well) into the 24-well plate, followed by cultivation with complete *ɑ*-MEM that contained RANKL, M-CSF, as well as HQSXD at specific doses for a 5-day period. After cell lysis, the TRAP activity assay kit was utilized for measuring TRAP activity in line with specific protocols. In brief, after removing the medium, PBS was used to rinse cells thrice, followed by 15-min lysis using a passive lysis buffer under 37°C. Thereafter, this work harvested supernatants for incubation using the para-nitrophenyl phosphate (p-NPP) for a 45-min period along with disodium tartrate. The sodium hydroxide solution was then added to terminate the reaction. The Infinite F200 PRO absorbance microplate reader (Tecan Group Ltd., Mannedorf, Switzerland) was employed to measure OD45 value for quantifying TRAP activity.

### 2.6. In Vitro Assay on Osteoclast Genesis

This work inoculated BMMs (3 × 10^6^/well) into the 24-well plate, followed by cultivation with complete *ɑ*-MEM that contained RANKL, M-CSF, as well as HQSXD at specific doses for a 5-day period with medium change at 2-day intervals. Later, cells were stained with TRAP by adopting the leukocyte acid phosphatase kit (Sigma-Aldrich) in line with specific protocols. Finally, the Olympus microscope (Waltham, MA, USA) was utilized to visualize TRAP-positive cells with multiple nuclei (>3) and to take photographs under 40× magnification.

### 2.7. Western Blot Assays

To conduct SDS-PAGE, a SDS lysis buffer was used to lyse BMMs-derived whole-cell lysates. Immunoblots were later analyzed using specific primary antibodies (1 : 1000) overnight under 4°C, followed by secondary antibody incubation with the Immobilon Western kit (Millipore, Billerica, MA, USA). Later, photographs were taken using the Image Quant LAS 500 imager (GE Healthcare, Waukesha, WI, USA). ImageJ (National Institutes of Health, Bethesda, MD, USA) was adopted for measuring protein levels.

### 2.8. Network Pharmacology (NP)-Based Analysis

#### 2.8.1. Data Collection

Chemical structure information of HQSXD was collected from the Traditional Chinese Medicine Systems database (https://tcmspw.com/tcmsp.php). In addition, osteoclastogenesis-related genes were identified from Gene Cards (https://www.genecards.org/), OMIM (https://omim.org/), and UniProt (https://www.uniprot.org/) with the keywords “osteoclast differentiation”, and “osteoclastogenesis”. The UniProt database was applied in confirming gene data like name as well as gene IDs.

#### 2.8.2. Network Construction and Topology Analysis

After importing the common targets into the STRING network platform (https://string-db.org/), the protein species was set to “Homo sapiens” and “Multiple proteins” was selected, followed by the highest interaction score (0.7). This work acquired network relation information regarding HQSXD-OC target interactions with the highest confidence level (0.7). The network relationship data were optimized for PPI network graphs using Cystoscape 3.8.8 software.

#### 2.8.3. Enrichment Analysis

This work utilized the R software clusterProfiler package for converting HQSXD-osteoclast target gene symbol into gene ID. Later, biological processes (BPs) as well as related pathways with significant differences in the intervention of HQSXD in osteoclast were selected by KEGG pathway analysis, upon the *P* < 0.05 threshold.

### 2.9. Statistical Analysis

Results were represented by mean ± SD from 3 separate assays. Differences were compared by one-way ANOVA or unpaired Student's *t*-test (two-sided). ^*∗*^*p* < 0.05, ^*∗∗*^*p* < 0.01 and ^*∗∗∗*^*p* < 0.001 stood for statistical significance.

## 3. Results

### 3.1. HQSXD Suppressed M-CSF-Mediated OC Formation with No Cytotoxic Effects

**Figure 1:**
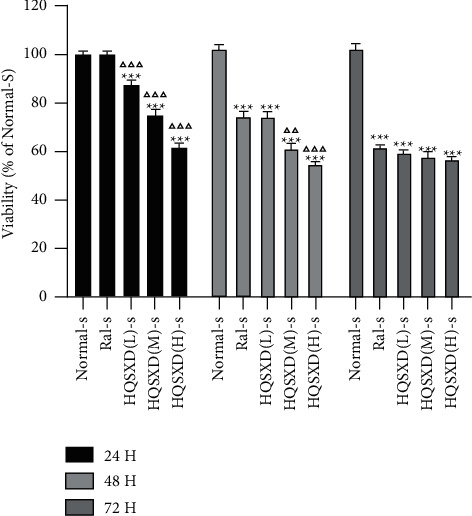
Effect of HQSXD on BMM proliferation. HQSXD at increasing doses was incubated with BMMs for 24, 48, and 72 h. Cell viability was then determined by CCK-8 assay.^*∗*^*P* < 0.05, vs. Normal-S; ^△^*P* < 0.05, vs. Ral-S; *n* = 6.

**Figure 2:**
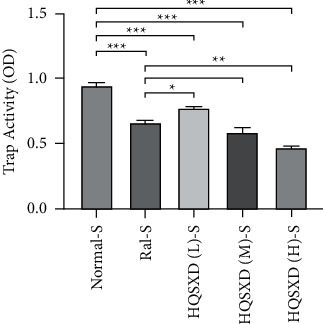
Effect of HQSXD on osteoclast precursor TRAP activity. HQSXD at the indicated concentrations was incubated with BMMs, the proliferation of which was sustained by M-CSF and RANKL for 5 days. Then, TRAP colorimetry was applied to identify the activity of osteoclast precursors. ^*∗∗∗*^*P* < 0.001, ^*∗∗*^*P* < 0.01, ^*∗*^*P* < 0.05; *n* = 6.

**Figure 3:**
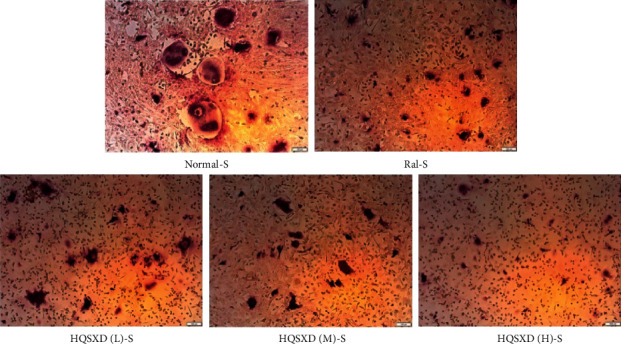
Effect of HQSXD on osteoclast differentiation. HQSXD at the indicated concentration was incubated with BMMs, which were induced into osteoclasts by M-CSF and RANKL. TRAP staining was applied to identify TRAP-positive multinucleated cells. Images are shown at 40× magnification.

**Figure 4:**
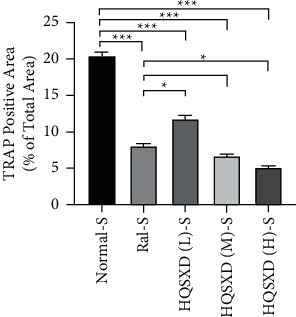
Calculated and graphical representation of area of TRAP-positive in Figure 3 multinucleated cells following different treatments. Calculation formula: area of positive cells/total field of view under the microscope; ^*∗*^*P* < 0.05; ^*∗∗∗*^*P* < 0.001; *n* = 3.

**Figure 5:**
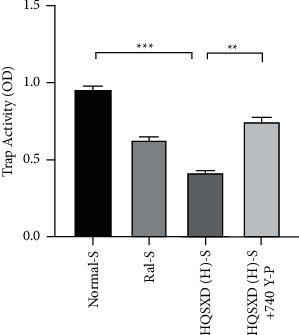
Effect of 740 Y-P intervention with HQSXD on TRAP activity of osteoclast precursors. HQSXD was incubated with BMMs at the indicated concentrations and the proliferation of BMMs was maintained by M-CSF and RANKL for 5 days. Concurrently, 740 Y-P was added for intervention. Then, TRAP colorimetric assay was applied to identify the activity of osteoclast precursors. ^*∗∗∗*^*P* < 0.001, ^*∗∗*^*P* < 0.01; *n* = 6.

**Figure 6:**
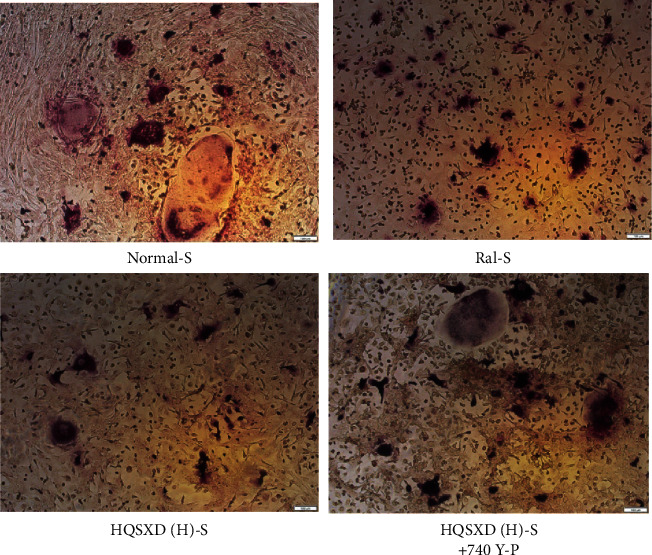
Effect of 740 Y-P intervention of HQSXD on osteoclast differentiation. HQSXD was incubated with BMMs at the indicated concentrations and the BMMs were induced to osteoclasts by M-CSF and RANKL. The 740 Y-P intervention was also added. TRAP staining was applied to identify TRAP-positive multinucleated cells. Images are shown at 40× magnification.

**Figure 7:**
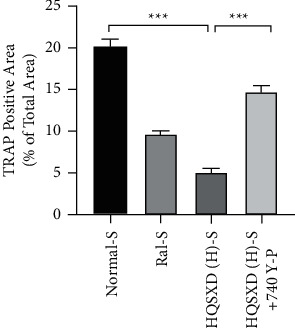
Calculated and graphical representation of area of TRAP-positive in Figure 6 multinucleated cells following different treatments. Calculation formula: area of positive cells/total field of view under the microscope; ^*∗∗∗*^*P* < 0.001; *n* = 3.

According to CCK-8 assay, HQSXD produced an inhibitory effect on BMMs (Figure 1). Additionally, HQSXD markedly suppressed OC's TRAP activity dose-dependently (Figure 2). For verifying HQSXD's inhibition on OC formation, mature OCs under the induction of M-CSF and RANKL along with HQSXD at specific doses were visualized by TRAP staining. Expectedly, control BMMs were induced differentiation to mature OCs, whereas HQSXD markedly suppressed TRAP-positive, multinucleated OC generation dose-dependently (Figures 3 and 4). This inhibition was effectively alleviated by the PI3K/Akt activator: 740Y-P (Figures 5–7). As a result, HQSXD suppressed BMM differentiation and activation, which was achieved via the PI3K/Akt pathway.

### 3.2. HQSXD Inhibited RANKL-Mediated OC-Related Protein Levels

**Figure 8:**
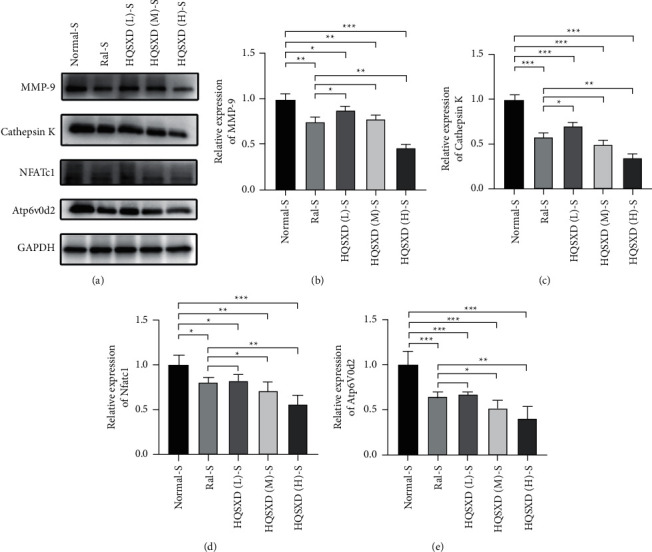
HQSXD inhibits RANKL-induced osteoclastogenesis-related protein expression in a dose-dependent manner. BMMs were stimulated with M-CSF and RANKL for 48 h in the presence of HQSXD; levels of MMP-9 (b), cathepsin K (c), NFATc1 (d), and Atp6v0d2 (e). Results were normalized to GAPDH expression. ^*∗∗∗*^*P* < 0.001, ^*∗∗*^*P* < 0.01, ^*∗∗*^*P* < 0.05; *n* = 3.

**Figure 9:**
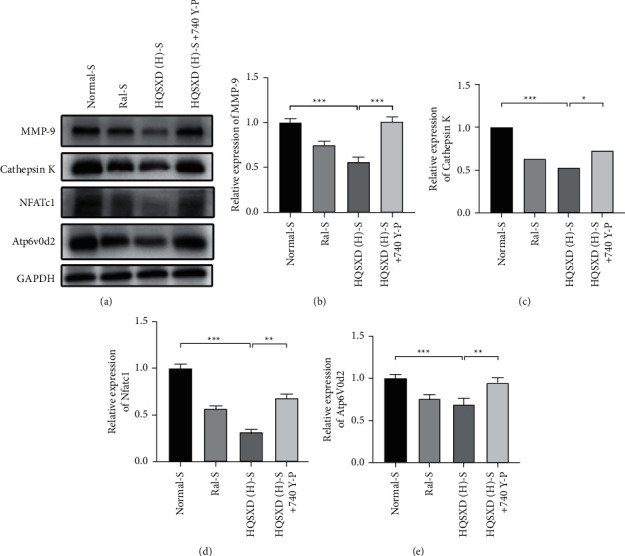
740 Y-P interferes with HQSXD to inhibit RANKL-induced osteoclastogenesis-related protein expression. BMMs were stimulated with M-CSF and RANKL for 48 h in the presence of HQSXD with the simultaneous addition of 740 Y-P; levels of MMP-9 (b), cathepsin K (CcNFATc1 (d), and Atp6v0d2 (e). Results were normalized to the expression of GAPDH. ^*∗∗∗*^*P* < 0.001, ^*∗∗*^*P* < 0.01, ^*∗∗*^*P* < 0.05; *n* = 3.

After 2-day HQSXD treatment at diverse doses, the OCs-related protein levels in OCs were analyzed. As a result, HQSXD markedly suppressed the expression of related proteins (MMP-9, cathepsin K, NFATc1, Atp6v0d2) dose-dependently (Figure 8). This inhibition was effectively alleviated by PI3K/Akt activator: 740Y-P (Figure 9). Based on the above findings, HQSXD suppressed the expression of proteins related to the differentiation process of osteoblasts, which was modulated via PI3K/Akt pathway.

### 3.3. Network Pharmacology Study

#### 3.3.1. Data Preparation

Based on the search of TCMSP and deriving the results, 189 active ingredients were obtained, and 170 active ingredients were obtained after removing duplicate ingredients. By searching the database, there were 3707 drug targets corresponding to the active ingredients of HQSXD, and 232 remained after removing duplicates. After searching the three databases with the keyword “osteoclast differentiation”, a total of 1835 disease targets were obtained, with 1823 remaining after removing duplicates. After searching the three databases with the keyword “osteoclastogenesis”, a total of 689 disease targets were obtained, with 686 remaining after removing duplicates. Subsequently, the targets of “osteoclast differentiation” and “osteoclastogenesis” were intersected to obtain 1858 total targets of osteoclast differentiation, of which 651 were intersected by the two keywords. There were 651 intersecting targets in the two keywords.

#### 3.3.2. Construction of the PPI Network and Identification of the Key Network

**Figure 10:**
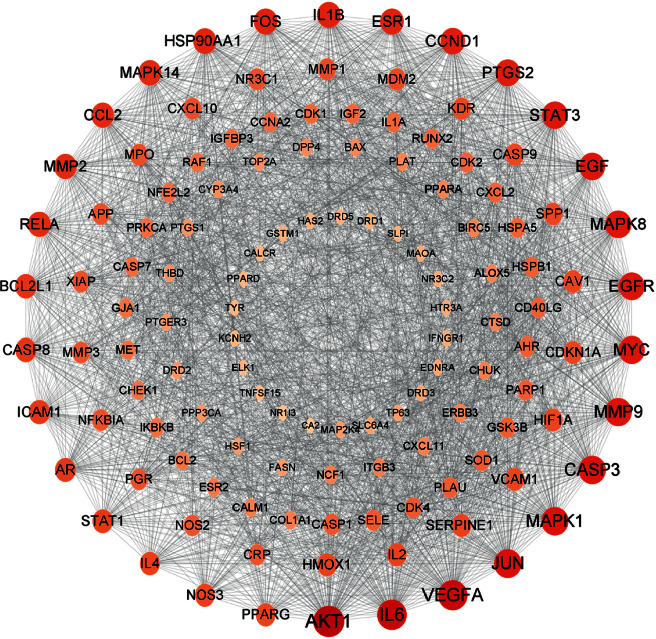
Results of the core PPI network. The core PPI network consists of 126 nodes and 4662 edges. The size of the nodes is arranged from the largest to smallest by degree, with a larger size indicating that the node is more “central”.

The PPI network between Huangqi Sanxian Decoction and osteoclast differentiation/production targets had 126 targets and 4662 interaction lines between the targets with an average degree of 37 (Figure 10).

#### 3.3.3. KEGG Signaling Pathway Analysis

**Figure 11:**
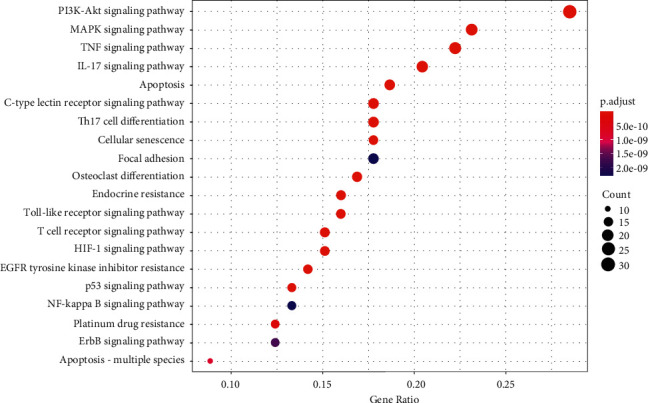
Results of the KEGG signaling pathway enrichment analysis. Bubble diagram of the first 20 KEGG signaling pathways.

**Figure 12:**
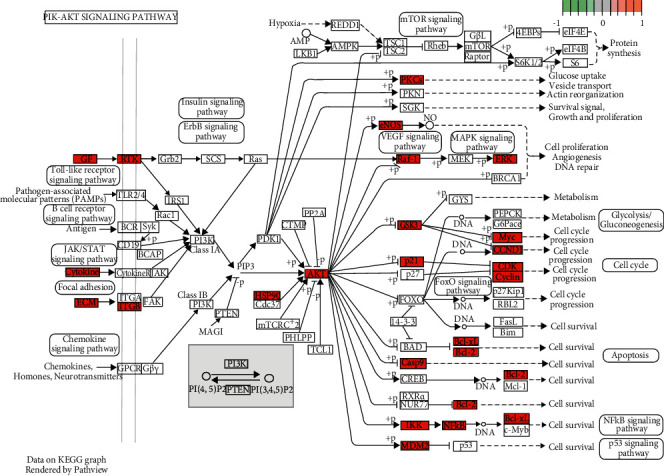
Enriched hsa04151 PI3K/Akt signaling pathway; predicted intervention pathway proteins are in red.

The abovementioned hub genes were conducted KEGG pathway analysis for revealing the candidate mechanism by which HQSXD affected OC formation, and 20 pathways were enriched (Figure 11). After literature retrieval, this work chose PI3K/Akt pathway (KEGG:04151), since it had great relativity whereas low *p*-value for OC formation (Figure 12).

### 3.4. HQSXD Mitigated RANKL-Triggered PI3K/Akt Pathway Activation

**Figure 13:**
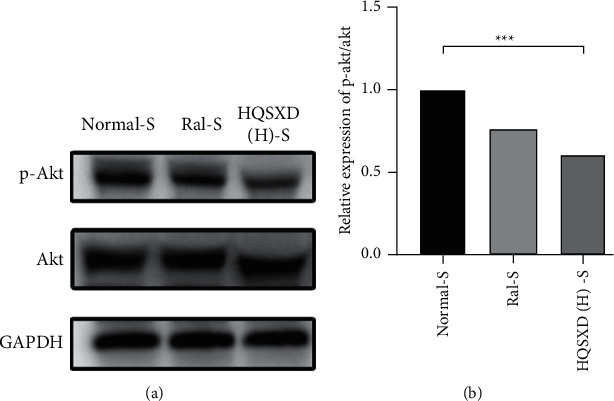
HQSXD impairs RANKL-induced Akt phosphorylation. (a) BMMs were pretreated with HQSXD for 2 day and then stimulated with RANKL for the indicated times. Cell lysates were then subjected to western blot analysis for p-Akt, Akt and GAPDH. (b) Quantification of p-Akt protein expression levels was normalized to total Akt levels. ^*∗∗∗*^*P* < 0.001; *n* = 3.

Based on KEGG analysis results, WB assay was conducted to analyze short-run protein phosphorylation mediated by RANKL, for the sake of verifying the mechanism of HQSXD in inhibiting OC generation and activity. BMMs were exposed to RANKL treatment with/without 2-day HQSXD pretreatment, and the phosphorylation of Akt was detected. Consequently, Akt phosphorylation elevated following RANKL treatment. Nonetheless, HQSXD pretreatment markedly suppressed phosphorylation of those aforementioned in comparison with controls (Figure 13).

## 4. Discussion

Bone accounts for the organ undergoing constant renewal and remodeling in one's life. OCs have been identified as the necessary part for maintaining bone health, since they have characteristic bone-resorption activity [18]. Actually, the existing agents used to manage osteopathic disorders mostly focus on bone resorption through modulating OC activity, apoptosis and differentiation [19]. Bisphosphonates have been commonly applied in the treatment of bone resorption clinically [20]. But they are still associated with certain side effects like mandibular osteonecrosis [21], atypical femoral fractures [22], as well as renal toxicity [23]. Great achievements have been attained by menopausal hormone therapy (MHT) for the treatment of postmenopausal osteoporosis (PMOP), but it also leads to increased risks of breast cancer (BC) endometrial cancer (EC), cardiovascular events, and blood clots [24,25].

This work evidenced that HQSXD suppressed the RANKL-mediated OC formation in vitro, and first revealed the underlying molecular mechanisms. Cell growth and viability have important effects on discovering agents against OC formation, since adverse reactions related to the frequently applied agents bisphosphonates are partially associated with their activity in OC viability [26,27]. As observed from Figures 1 and 2, HQSXD produces an inhibitory effect on BMMs. Additionally, TRAP staining and WB assays were conducted to analyze HQSXD's impact on OC differentiation. As a result, HQSXD totally suppressed the differentiation of BMMs to mature OCs with TRAP-positivity (Figures 3 and 4). Furthermore, the RANK/RANKL pathway is identified to be an important pathway for regulating OC genesis and absorption. NFATc1 is a TF with strongest induction after RANKL treatment, which modulates many OC-related proteins like cathepsin K, TRAP, MMP-9, and Atp6v0d2 [28]. HQSXD treatment inhibits the role of proteins associated with the differentiation of BMMs (Figure 8).

Natural medicines can synergistically affect several targets, components as well as pathways [29]. The complexity has added to the difficulty in analyzing components with pharmacological activity as well as natural products' molecular mechanism. NP, first put forward by the British pharmacologist Hopkins [30], has been extremely appropriate for illustrating natural products' potential in treatment and mechanism at molecular level [31]. For investigating the mechanism of HQSXD in inhibiting OC differentiation, hub targets obtained from core PPI network were conducted enrichment analysis (Figures 10 and 11).

Combining the core PPI network with the results of the enrichment analysis, after reviewing the literature, the PI3K/Akt pathway was determined to be the possible molecular mechanism related to HQSXD's role in suppressing OC formation. The PI3K/Akt signaling is frequently involved in multiple signaling molecules in osteoclasts, including Akt, GSK3*β,* and CCL2, which are all in the core PPI network (Figure 10). CCL2, as an upstream signaling factor of the PI3K/Akt signaling pathway, activates the PI3K/Akt signaling pathway upon binding to its most potent receptor CCR2 [32]. GSK3*β* as a downstream signaling factor of PI3K/Akt signaling pathway can regulate NFATc1 expression, and the PI3K/Akt/GSK3*β*/NFATc1 signaling pathway is important in osteoclast differentiation and formation [33].

Therefore, we selected Akt, a core protein in the PI3K/Akt signaling pathway, for a WB assay to test the above hypothesis. As a result, HQSXD remarkably suppressed Akt's short-time phosphorylation (Figure 13). For better verifying PI3K/Akt pathway's effect on the suppressed OC formation by HQSXD, this work found that, 740-Y-P, the activator of PI3K/Akt pathway, could alleviate HQSXD treatment after being inhibited in TRAP-positive mature osteoblasts (Figures 5–7 and 9). More importantly, the PI3K/Akt signaling pathway activator (740-Y-P) could attenuate the effect of HQSXD treatment that inhibited proteins associated with the differentiation of BMMs. In addition, Akt, the core factor of PI3K/Akt signaling pathway, and NFATC1, the transcription factor of osteoclast differentiation, were both inhibited under the intervention of Huangqi Sanxian decoction.

Therefore, we suggest that Huangqi Sanxian Tang can inhibit osteoclast differentiation by inhibiting PI3K/Akt signaling pathway, and the network pharmacology results show that CCL2 and GSK3*β* are most likely the upstream and downstream signaling factors that exert their inhibitory effects. This is the first study demonstrating HQSXD's inhibition on RANKL-induced OC differentiation into BMMs as well as the related mechanisms. It might help to interpret the molecular mechanism related to HQSXD within OC-associated disorders. Moreover, NP analysis was consistent with molecular mechanism study, which indicated the possibly of NP in modernizing TCM.

## 5. Conclusion

In this work, HQSXD shows toxicity to OBs and inhibits osteoclast differentiation. The inhibitory effect was correlated with the PI3K/Akt signaling pathway and the network pharmacology results show that CCL2 and GSK3*β* are most likely the upstream and downstream signaling factors that exert their inhibitory effects. Further studies are needed to investigate how HQSXD exerts its effect on osteoclast differentiation via suppressing the PI3K/Akt pathway and thus osteoclast differentiation.

## Data Availability

The data used to support the findings of this study are included within the article.
